# Disease resistance is related to inherent swimming performance in Atlantic salmon

**DOI:** 10.1186/1472-6793-13-1

**Published:** 2013-01-21

**Authors:** Vicente Castro, Barbara Grisdale-Helland, Sven M Jørgensen, Jan Helgerud, Guy Claireaux, Anthony P Farrell, Aleksei Krasnov, Ståle J Helland, Harald Takle

**Affiliations:** 1 Nofima, Ås, Norway; 2 Institute of Animal Sciences, Norwegian University of Life Sciences (UMB), Ås, Norway; 3 AVS Chile S.A., Puerto Varas, Chile; 4 Aquaculture Protein Centre, CoE, Ås, Norway; 5 Nofima, Ås, Norway; 6 Norwegian University of Science and Technology, Faculty of Medicine, Trondheim, Norway; 7 Université de Bretagne Occidentale, LEMAR, Unité de Physiologie Fonctionnelle des Organismes Marins, Ifremer, Plouzané, France; 8 Faculty of Land and Food Systems, & Department of Zoology, University of British Columbia, Vancouver, BC, Canada

## Abstract

**Background:**

Like humans, fish can be classified according to their athletic performance. Sustained exercise training of fish can improve growth and physical capacity, and recent results have documented improved disease resistance in exercised Atlantic salmon. In this study we investigated the effects of inherent swimming performance and exercise training on disease resistance in Atlantic salmon.

Atlantic salmon were first classified as either poor or good according to their swimming performance in a screening test and then exercise trained for 10 weeks using one of two constant-velocity or two interval-velocity training regimes for comparison against control trained fish (low speed continuously). Disease resistance was assessed by a viral disease challenge test (infectious pancreatic necrosis) and gene expression analyses of the host response in selected organs.

**Results:**

An inherently good swimming performance was associated with improved disease resistance, as good swimmers showed significantly better survival compared to poor swimmers in the viral challenge test. Differences in mortalities between poor and good swimmers were correlated with cardiac mRNA expression of virus responsive genes reflecting the infection status. Although not significant, fish trained at constant-velocity showed a trend towards higher survival than fish trained at either short or long intervals. Finally, only constant training at high intensity had a significant positive effect on fish growth compared to control trained fish.

**Conclusions:**

This is the first evidence suggesting that inherent swimming performance is associated with disease resistance in fish.

## Background

Diseases represent the main constraint for the success of an expanding aquaculture industry. Atlantic salmon (*Salmo salar*) farmers can experience severe fish losses due to both infectious and non-infectious diseases, usually during the seawater growth stage. Infectious pancreatic necrosis (IPN), pancreas disease (PD), infectious salmon anemia (ISA), as well as the sea lice parasite (*Lepeophtheirus salmonis* K) represent some of the most hazardous diseases [1,2], but losses have also been associated with non-infectious diseases such as cardiac failures [3,4]. Biosecurity countermeasures to control the disease situation include vaccines and pharmaceuticals, as well as improvements of the genetic material, feeds and husbandry practices. The aim of current and future countermeasures is to strengthen the fish robustness, which is the capability to combine fast growth and normal organ development with improved resistance to both disease and physiological challenges.

Sustained exercise training has been documented to confer higher robustness to cultured fish, including increased somatic and cardiac growth, cardiac performance, aerobic capacity of the muscle, oxygen carrying and extraction capacity and improved bone quality [5-10]. Khovanskiy et al. [11] found that exercised chum salmon (*Oncorhynchus keta*) displayed lower mortality associated with an improved osmoregulatory capacity after seawater transfer when compared to untrained fish. Going further, we have recently demonstrated direct effects of sustained exercise on disease resistance, showing that survival of Atlantic salmon challenged with infectious pancreatic necrosis virus (IPNV) was 13% higher for fish subjected to a moderate interval-training regime for six weeks prior to smoltification when compared with fish held at a low, constant swimming speed [12]. Thus, sustained exercise in fish can induce a similar robustness effect as in humans, where a moderate aerobic training is also known to decrease the risk of infections and chronic life-style diseases [13,14].

It has been observed that exercised salmonids [12,15] and non-salmonids, (*Plecoglossus altivelis*[16]; *Chalcarburnus chalcoides mento*[17]; *Morone saxatilis*[18]; *Sparus aurata*[19]; *Danio rerio*[20]) exhibit improved growth due to improved feed efficiency, higher feed intake or a mix of both. Several studies have reported on the relationship between improved growth performance and disease resistance in fish (see reviews by Merrifield et al. [21,22]). For example, Gjedrem [23] suggested that a breeding program selecting for growth also induced a positive genetic response for disease resistance, although conflicting results exist [24]. Recently, an association between exercise-induced growth and improved disease resistance was shown in Atlantic salmon [12]. The possibility of a linkage between these two factors, growth and disease resistance, is of obvious importance for the fish farming sector.

Migratory fish such as salmonids have a great inherent capacity for sustained aerobic swimming. Benefits from exercise seem to be maximized at speeds close to the optimal swimming speed (U_opt_), where energy use is more efficient and the cost of transport is minimized [25]. Exercising fish at speeds other than U_opt_ results in additional energy usage for locomotion, even at low speeds due to behavioral changes (e.g. increased aggression and spontaneous activity). Further, the highest speeds may prove stressful and unsustainable compared with U_opt_[20]. Because of their natural swimming behavior and high aerobic capacity, salmonids are naturally amenable to long-term continuous exercise training, provided sustainable water velocities are used. This is in contrast to terrestrial animals that more typically require resting periods between bouts of exercise training. In humans, where most exercise training research has been performed, the intensity seems to be a fundamental factor affecting the individual’s systemic immunity. While engaging in regular moderate exercise activity seems to enhance immune functions [13], high intensity aerobic training results in acute and chronic states of impaired immunity [26].

On top of training effects, there seems to be an equally large inherent variation in exercise capacity among fish and humans. For example, juvenile rainbow trout (*Oncorhynchus mykiss*) can be classified according to their inherent swimming performance as either poor or good swimmers. Interestingly, such classification was associated with several cardiac and metabolic capacities after 9 months of common rearing [27].

This study aimed to evaluate if inherent swimming performance in juvenile fish affect disease resistance. Juvenile fish were identified according to their inherent swimming performance by pre-screening them in a swim challenge test. Fish classified as either poor or good swimmers were then trained at four different regimes to investigate if training differentially affected them. After smoltification, a controlled disease challenge with IPNV allowed us to assess differences in disease resistance among and within the two performance groups and four training regimes. This was further examined by analyzing expression levels of sensitive virus responsive genes (VRGs) in head kidney and cardiac tissues. In addition, exercise-induced effects on robustness were evaluated by growth performance and feed efficiency.

## Results

### Disease resistance is related to inherent swimming performance

**Figure 1:**
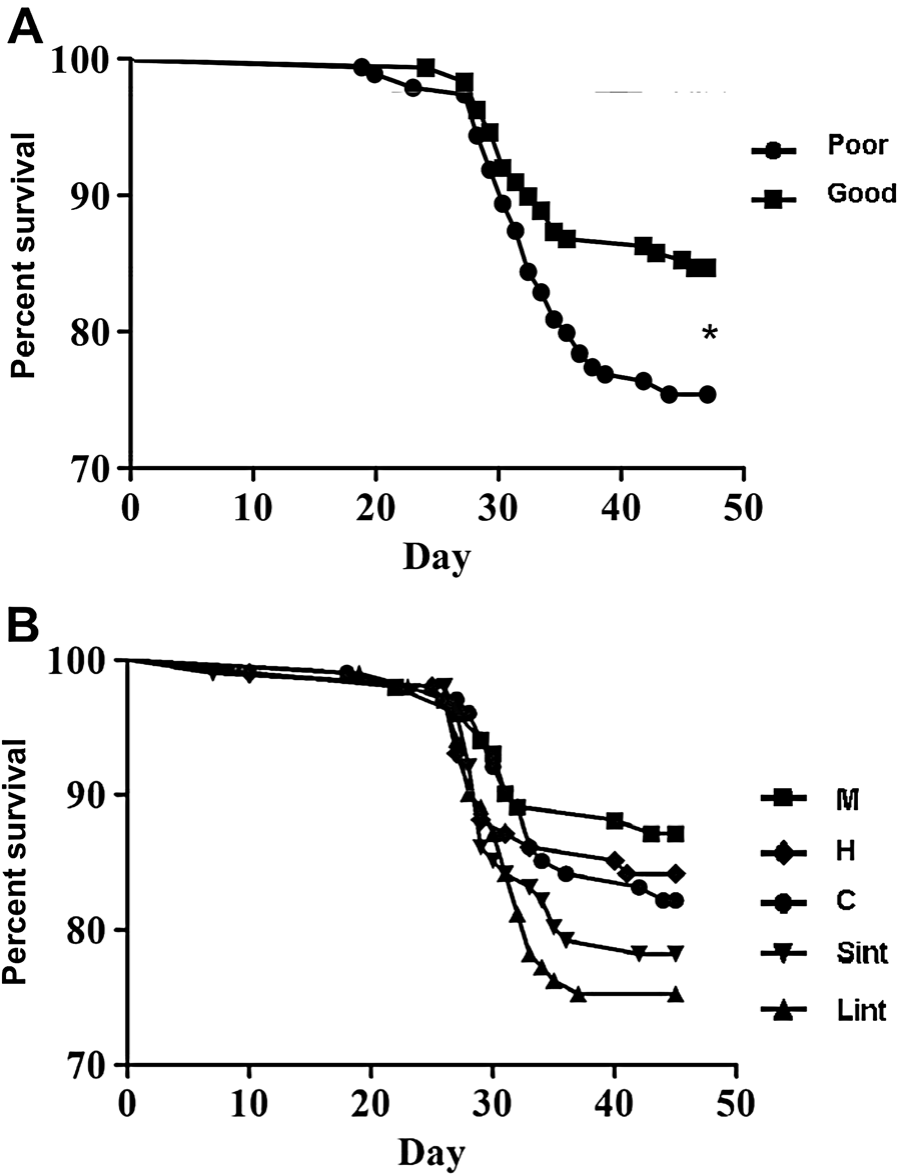
**Cumulative survival of swimming performance and exercised groups during IPN challenge. A**: The inherent swimming performance of the fish had a significant effect on disease resistance, with the good swimmers showing a higher survival (86.1%) than the poor swimmers (77.6%). **B**: Fish exercised at constant speeds for 10 weeks showed a trend towards higher disease resistance compared to those subjected to interval training regimes (Medium intensity (M) = 87.1%; High intensity (H) = 84.2%; Control (C) = 82.2%; Short interval (Sint) = 78.2% and Long interval (Lint) = 75.3%) (p = 0.21). *indicates significant difference, Mantel-Cox test; p < 0.05.

Mortality following the IPN challenge started 18 days after the introduction of virus-shedding fish and reached a plateau around day 38 post-challenge. Inherent swimming performance showed a strong association with survival after the IPN challenge test. Fish initially categorized as good swimmers had significantly better survival than poor swimmers (86.1 and 77.6%, respectively; p = 0.02) when analyzed across all groups (Figure 1A). When survival was examined independent of the inherent swimming performance, differences among training regimes showed no significant difference (p = 0.21). The continuous-velocity training regimes tended to improve survival compared to the control trained group (87.1, 84.2 and 82.2% survival for respectively M, H and C), whereas interval-velocity training regimes tended to negatively impact survival (78.2 and 75.3% survival for Sint and Lint, respectively; Figure 1B).

**Figure 2:**
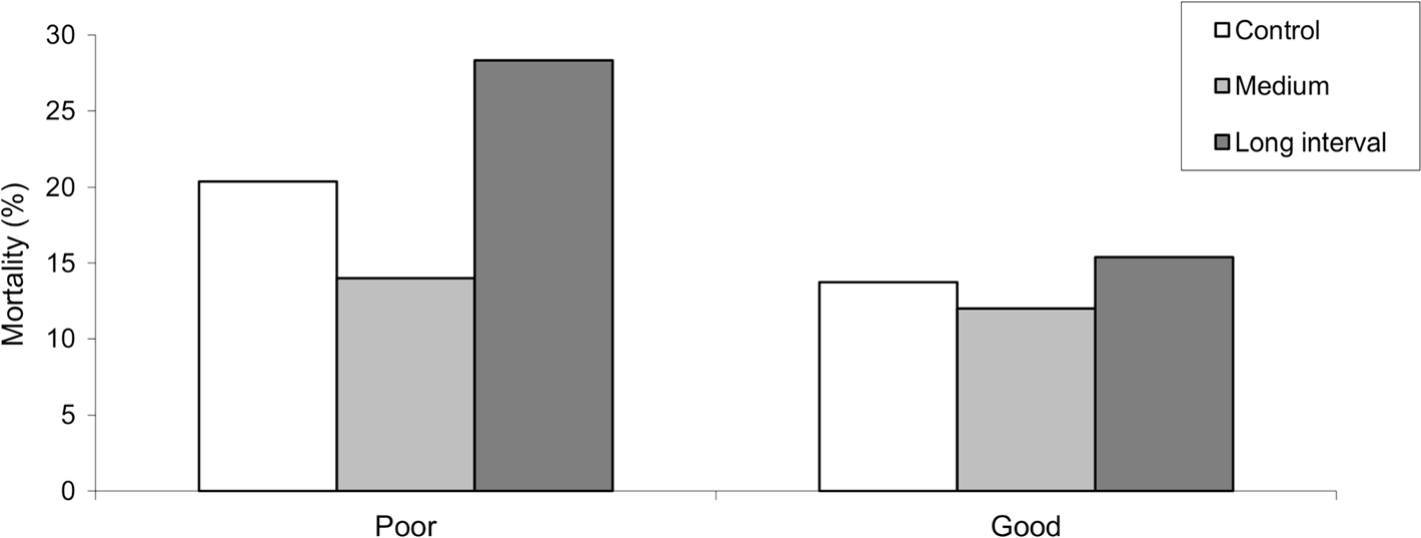
**Interaction between inherent swimming performance and exercise training on disease resistance.** Only those regimes included in the gene expression analysis are displayed. These reflect the lower (Medium), middle (Control) and higher (Long interval) mortalities found in response to IPN infection. Data is shown within poor (left) and good (right) swimmers. Though not significant, differences in mortalities were larger between poor and good swimmers for the Long interval, smaller for Control and minimum for the Medium intensity regime. Initial number of fish on the challenge ranged from 100 to 112 per regime.

When survival was examined in light of swimming performance, exercise training did not significantly affect disease resistance of poor and good swimmers. Nevertheless, exercise may have had a larger impact on disease resistance of poor swimmers since larger changes in mortality were observed in exercised groups of poor swimmers compared to good swimmers (Figure 2).

### Virus responsive gene expression reflects and supports mortality data

To verify mortality data from IPNV, infection level was analyzed in challenged fish at termination of the disease trial. Quantification of IPNV (by real-time qPCR in head-kidney) in surviving fish showed low prevalence of virus-positive fish (33%) and low level of viral transcripts in positive fish. Heart tissue was also tested and found negative for all fish. Thus, sampled fish were in a late stage of infection with either low levels or no virus replication.

**Figure 3:**
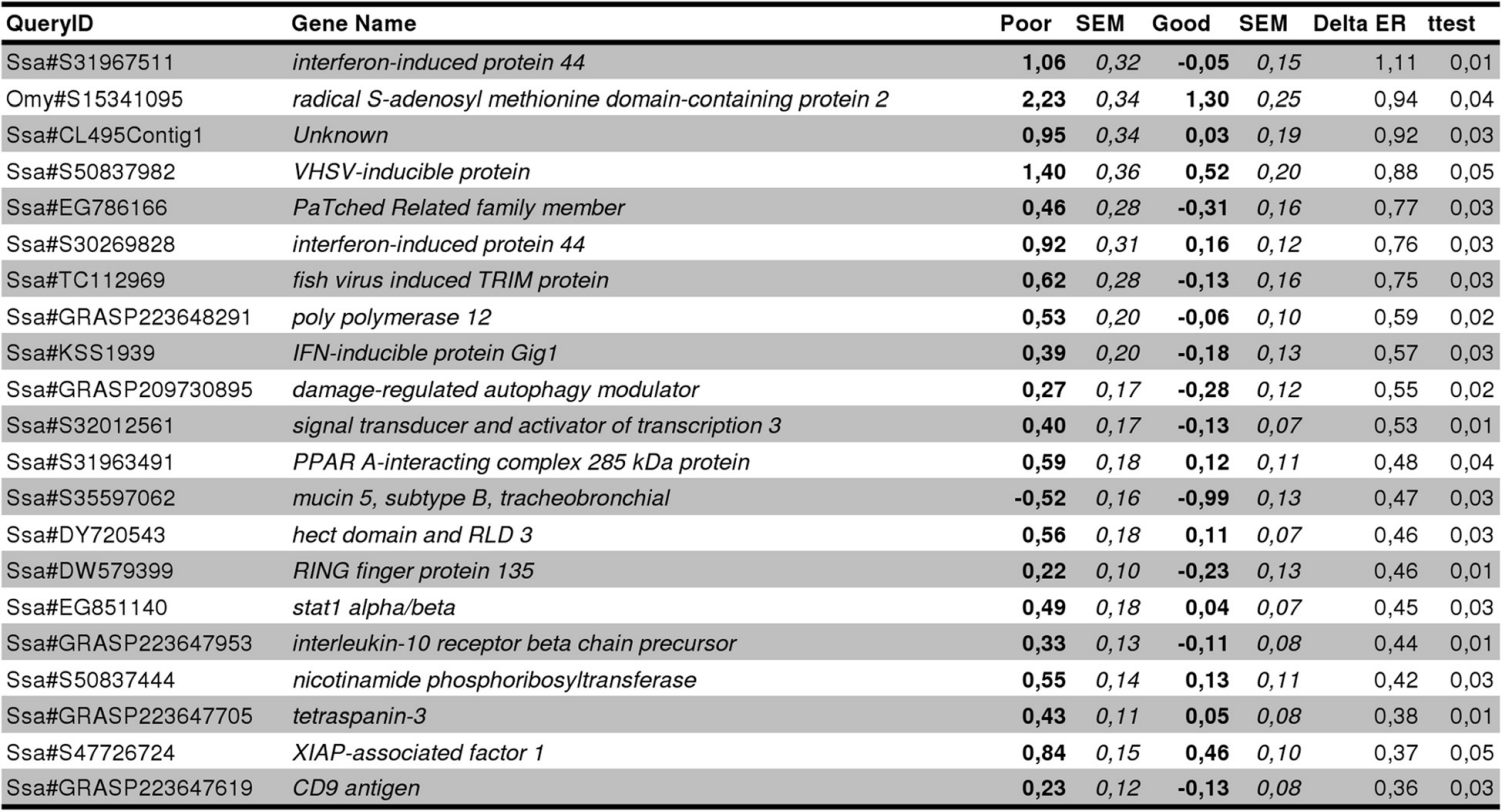
**Differentially expressed genes in poor versus good swimmers post IPN challenge.** Microarray analyses resulted in 21 genes showing higher transcript abundance in cardiac muscle of poor swimmers compared to good swimmers. By function, all of these genes have been previously identified as virus responsive genes (VRGs). Data for Poor and Good swim performance groups is log_2_-ER (expression ratio) ± SEM (n = 9).

Gene expression analysis was performed to investigate among-groups differences in host immune correlates of disease response. For initial screening of correlates, transcriptome analysis by microarray of poor and good swimmers was assessed, since the strongest contrast in mortality was associated with swimming performance. This resulted in 21 genes with significantly higher transcript abundance in poor compared to good swimmers (*t*-test, p < 0.05) (Figure 3). By function, all genes were previously identified as virus-responsive genes (VRGs); a group of genes displaying a common activation/transcription to most of the known viruses infecting Atlantic salmon. VRGs are sensitive antiviral markers reflecting the infection status and the level of viral transcripts in cells [28]. Thus, poor swimmers seemed to have higher activation of antiviral immune genes compared to good swimmers at the end of the infection trial.

**Figure 4:**
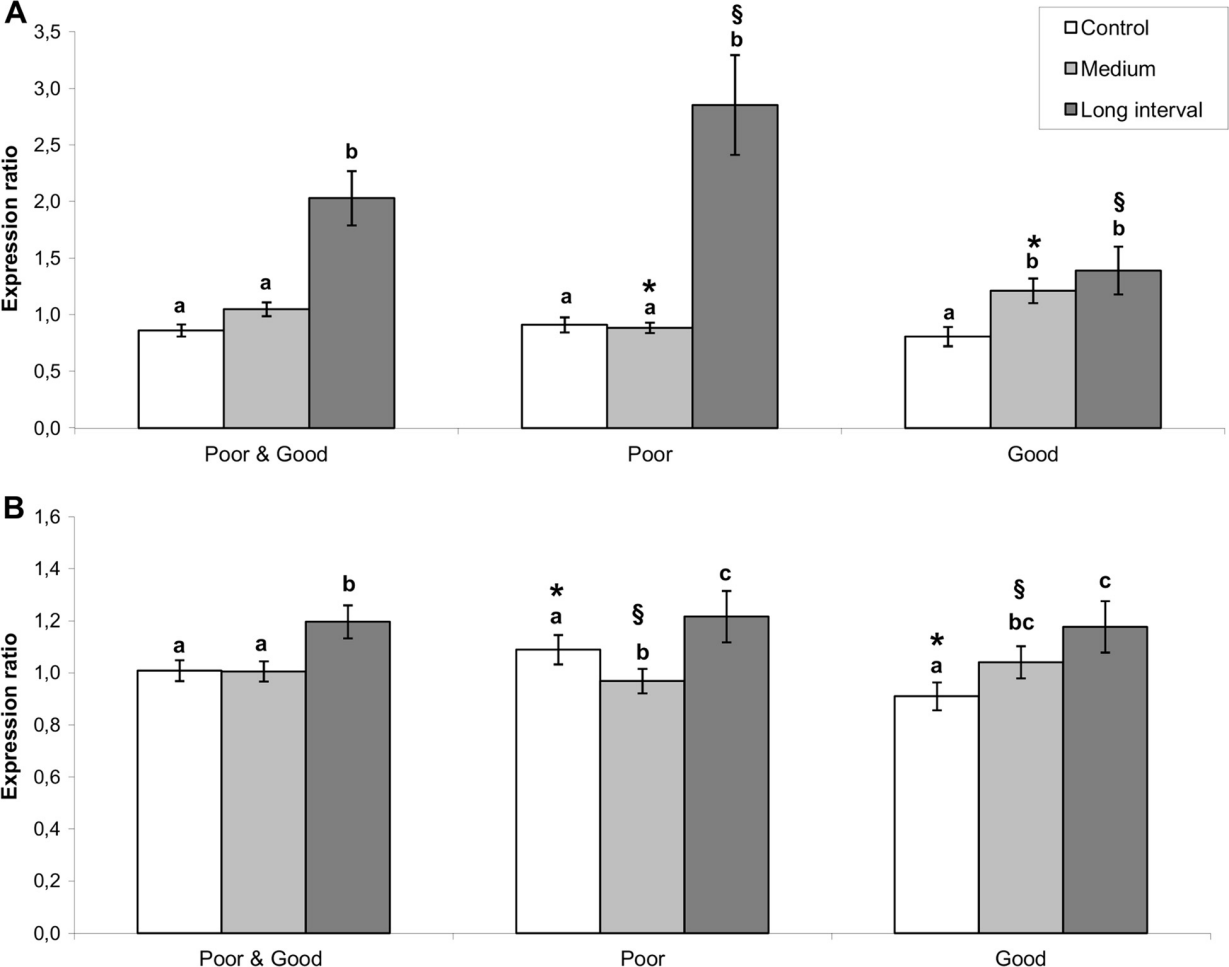
**Expression of virus-responsive genes in tissues of exercised and swim performance groups post IPN challenge.** Improved survival after IPN challenge was associated with the expression of virus responsive genes (VRGs) as assessed by qPCR. Higher expression levels of VRGs in cardiac tissue of poor compared to good swimmers from the Lint regime (**A**), and in head kidney of poor compared to good swimmers from the control group (**B**) reflected the overall higher mortality of poor swimmers in comparison to the good swimmers. Further, VRGs expression in both tissues was in concordance with differences in survival between interval (Lint) and constant speed (Control and Medium) training regimes. Bars represent expression ratio ± SEM relative to a pooled control sample and normalized against two reference genes (*18S rRNA* and *elongation factor 1α*) with correction for PCR efficiency. For **A** and **B**, each bar is a composed index of 6 (n = 8 fish/swim-performance/regime) and 8 (n = 5 fish/swim-performance/regime) VRGs, respectively. Genes included: RSAD2 (*radical S-adenosyl methionine domain containing protein2*) (A + B), IFIT5 (*interferon-induced protein with tetratricopeptide repeats 5*) (A + B), STAT1 (*signal transducer and activator of transcription 1*) (A + B), VHSV2 (*viral haemorrhagic septicaemia virus-inducible protein*) (A + B), BAF (*barrier-to-autointegration factor*) (A + B), GIG1 (i*nterferon-inducible Gig1*) (A + B), RIG-I (*DEAD/H* (*Asp-Glu-Ala-Asp/His) box polypeptide*) (**B**), MDA5 (*interferon induced with helicase C domain 1*) (**B**). abc: Denotes significant difference (p < 0.05; paired *t*-test) between training regimes. Other symbols (*§) denotes significant difference (p < 0.05; paired *t*-test) between poor and good swimmers within each training regime.

To further substantiate these results and to evaluate effects of the different training regimes with sufficient biological replication, expression of six VRGs was analyzed in heart ventricle tissue of poor and good swimmers from the C, M and Lint exercise-trained groups using qPCR. Results showed that induced levels of VRGs in poor swimmers were mainly explained by a strong expression in Lint exercised fish (Figure 4A). Control trained (C) and M trained poor swimmers had equal expression level of VRGs. Within the good swimmers, VRG expression levels were higher in M and Lint trained compared to control trained fish.

We further analyzed expression of eight VRGs in head kidney, where IPNV replication was observed. Expression levels within exercised good swimmers showed a similar trend as observed for heart tissue (Figure 4B). Within poor swimmers, VRG expression levels were significantly lower in M compared to both control and Lint trained fish (p < 0.05). In contrast to heart tissue, VRG expression levels in head-kidney of control trained good swimmers were significantly lower compared to control trained poor swimmers (p = 0.03). Thus, the increased overall mortality observed for poor swimmers was reflected by stronger expression levels of antiviral immune genes in Lint trained (heart) and control trained (head-kidney) fish as compared to good swimmers. While no evidence for any positive effects of exercise training on mortality and infection status was observed for good swimmers, results implied beneficial effects in terms of reduced infection status (lower VRG expression) from M trained poor swimmers.

Results produced for 8 genes by microarray and qPCR were in close concordance (Pearson *r* = 0.92; p = 0.001).

### Exercise training effects on growth

After six weeks of exercise training, no significant differences in thermal growth coefficient (TGC) were found among training regimes and control trained fish. At the end of the freshwater phase of the experiment (10 weeks of training plus 1 week detraining), TGC was significantly higher (p < 0.05) in the high intensity (H) training regime (1.61) compared with the control (C) trained group (1.50) (Table 1). The other training regimes only showed a trend towards higher TGC compared to the control trained group (p < 0.1). At the end of exercise training, medium intensity (M) trained fish showed a significantly higher condition factor (CF) than C. Feed intake showed significant differences among training regimes, with the higher values belonging to the H and short interval (Sint) regimes at the end the first six training weeks, while H and long interval (Lint) had the highest feed intake in the second part of the training experiment. After six weeks of training, the only significant differences for feed efficiency ratio (FER) among training groups were H and Sint groups being lower than both C and M (Table 1).

**Table 1:** Growth parameters and dry matter intake of exercise trained Atlantic salmon

		**C**	**M**	**H**	**Sint**	**Lint**
Body weight (BW) (g)	Start	40.9 ± 0.2	40.7 ± 0.2	40.6 ± 0.2	40.2 ± 0.4	41.0 ± 0.4
	W 6	70.5 ± 1.3	70.9 ± 0.7	72.4 ± 2.4	72.3 ± 0.6	71.4 ± 0.4
	W 11	95.4 ± 0.3	99.9 ± 1.1	99.4 ± 2.4	98.2 ± 2.2	100.4 ± 2.2
Length (cm)	Start	15.1 ± 0.03	15.0 ± 0.04	15.0 ± 0.01	15.0 ± 0.05	15.1 ± 0.03
	W 11	20.0 ± 0.04	20.2 ± 0.07	20.2 ± 0.16	20.2 ± 0.14	20.4 ± 0.14
CF	Start	1.18 ± 0.003	1.18 ± 0.002	1.18 ± 0.005	1.18 ± 0.002	1.18 ± 0.001
	W 11	1.18 ± 0.004^b^	1.20 ± 0.004^a^	1.19 ± 0.004^ab^	1.18 ± 0.002^b^	1.18 ± 0.008^b^
TGC	W 1-6	1.56 ± 0.05	1.59 ± 0.04	1.66 ± 0.09	1.68 ± 0.02	1.59 ± 0.01
	W 6-11	1.44 ± 0.08	1.64 ± 0.06	1.53 ± 0.05	1.47 ± 0.12	1.64 ± 0.10
	W 1-11	1.50 ± 0.01^b^	1.58 ± 0.03^ab^	1.61 ± 0.02^a^	1.59 ± 0.05^ab^	1.59 ± 0.03^ab^
Relative feed intake (% BW d^-1^)	W 1-6	0.87 ± 0.02^c^	0.88 ± 0.02^c^	0.98 ± 0.03^ab^	0.99 ± 0.01^a^	0.92 ± 0.01^bc^
	W 6-11	0.65 ± 0.02^c^	0.69 ± 0.01^bc^	0.74 ± 0.01^a^	0.70 ± 0.02^ab^	0.74 ± 0.01^a^
FER	W 1-6	1.43 ± 0.04^a^	1.42 ± 0.02^a^	1.32 ± 0.00^b^	1.34 ± 0.00^b^	1.37 ± 0.02^ab^
	W 6-11	1.66 ± 0.08	1.70 ± 0.03	1.52 ± 0.06	1.56 ± 0.11	1.58 ± 0.04

*C* Control, *M* medium intensity, *H* high intensity, *Sint* short interval, *Lint* long interval, *CF* condition factor, *TGC* thermal growth coefficient, week (W) 6: End of first six weeks of training under a short day light photoperiod. W 11: End of 10 weeks of training and one week of detraining. Means in the same row with different superscripts letters are significantly different based on one-way ANOVA (p < 0.05). Differences between the groups were assessed by the least-squares means procedure. Data are means ± SEM.

## Discussion

In this study we found that the inherent swimming performance of juvenile Atlantic salmon is associated with differences in survival to an infectious disease challenge after seawater transfer, with good swimmers displaying a significantly higher disease resistance than poor swimmers. Exercise training had no significant effect on disease resistance, though a trend towards improved performance was seen for fish being trained at constant compared to interval regimes. Though not significant, results further argue for exercise training affecting poor swimmers more strongly than good swimmers. Mortalities were supported by mRNA expression levels of a subset of VRGs reflecting the antiviral response status. Growth was promoted by exercise training, though only significant for the highest intensity regime (H), while swimming performance did not show an association with growth performance.

### The impact of inherent swimming performance on disease resistance

The inherent swimming performance of individual juvenile salmon was positively associated with disease resistance. Fish classified as good swimmers showed 8.5% better survival against IPN virus than poor swimmers when challenged 3 months after the swimming performance classification. Claireaux et al. [27] demonstrated that good swimmers of a cohort of rainbow trout, classified by a similar methodological approach as used in this work, retained this advantage nearly nine months later, despite a common rearing environment and similar growth performance, and displayed a significantly better cardiac capacity and morphology compared to poor swimmers. This and the present study collectively suggest that a simple screening test for swimming performance can efficiently distinguish between fish with low and high robustness, with the latter possessing better cardiac capacity and disease resistance. It must be noted that none of these studies could find differences in growth performance between poor and good swimmers.

In addition to the effects of the inherent swimming performance of fish on robustness, exercise training appeared to have a stronger, though not significant, modulatory effect on the poor swimmer’s disease resistance. While the M training regime showed a tendency to improve the survival rate of the poor swimmers, the Lint regime tended to decrease the survival of the poor swimmers. The possible interaction effect between inherent poor swimming performance and training regime on survival was supported by expression analysis of VRGs in surviving fish from the different training regimes and performance groups at the end of the IPN challenge. Results showed that improved survival of good swimmers was associated with lower expression levels of virus responsive genes, probably reflecting an overall lower level of infection pressure, a more rapid or efficient viral clearance and/or a reduced antiviral status to recover and regain homeostasis. Thus, the ability to rapidly clear or reduce virus replication and antiviral immunity at the end of a viral infection might be important for survival. In a previous study, we demonstrated that the improved survival induced by sustained training of juvenile Atlantic salmon, was related to a specific cardiac transcriptome signature, suggesting lower levels of inflammation and higher levels of immune effector molecules, antioxidant enzymes and xenobiotics clearance capacity prior to an IPN challenge [12].

### The impact of training regimes on disease resistance

Overall, the three continuous training regimes (including C) displayed a trend, though not significant, towards higher survival compared to the interval training regimes. The continuous 0.65 body lengths (BL)s^-1^ M regime gave the best results, which is in agreement with our previous finding where Atlantic salmon pre-smolts trained at a similar intensity for six weeks (0.8 BLs^-1^ for 16 h and 1 BLs^-1^ for 8 h per day) showed 13% higher survival following an IPN challenge test when compared to control trained fish [12]. Such improvements are very important in an industry context. In contrast to the improvement in disease resistance from utilizing an interval training regime with mild speed changes as in the previously mentioned study, the ~3-fold daily changes in swimming speed applied for the Sint and Lint regimes tended to reduce disease resistance against IPN compared to controls kept continuously at 0.32 BLs^-1^. Since Sint and Lint trained fish had theoretically swum the same distance as the M trained fish, it could be argued that the relatively strong daily changes in water speed for both interval regimes may be the cause of the apparent negative impact on disease resistance of these fish.

We may speculate that the non-significant trend towards reduced disease resistance of the inherently poor swimmers trained with the Lint regime is due to a lower acclimation capacity of these fish to the relatively strong changes in swimming velocity compared to the good swimmers. It is then plausible to suggest that poor swimmers suffered from higher stress levels when following the Lint compared to the continuous speed regimes, which could potentially cause an impairment of their disease resistance capacities. Inherently good swimmers, however, seem to have sufficient behavioral and/or physiological plasticity as to avoid disease resistance impairment.

The moderate intensity of the M training regime may have promoted an acclimative response in the poor swimmers, boosting their disease resistance to the level of the good swimmers. Thus, if confirmed with new studies, the overall profit of conducting M regime training would be the achievement of a more homogenous population when it comes to disease resistance. This would imply an indisputable benefit for salmon producers.

### The impact of training regimes on growth and feed utilization

By definition, a robust fish must possess a good combination of both high growth performance and disease resistance. Jobling et al. [29] and Davison [15] stated that higher growth may be achieved for fish when training intensity lies between 0.75 and 1.5 BLs^-1^. Our results are in agreement with this; the H training regime (1.31 BLs^-1^) had significantly higher TGC than the control trained group. Interestingly, the other three regimes (M, Sint and Lint), which had an average water speed of 0.65 BLs^-1^, showed a tendency towards improved growth compared to control trained fish, suggesting the existence of a correlation between growth and total work load of the swimming-induced exercise. Higher growth given by the H training regime was mainly due to increased feed intake associated with a lower feed efficiency and protein retention. This suggests that fish subjected to that regime required more energy to satisfy the increased demand. Despite a lowered feed efficiency, increases in feed intake were sufficient to overcompensate the needs of simultaneous swimming and growth. It could be argued that training at this intensity stimulated the regulation of neuroendocrine factors involved in controlling feeding, resulting in an anabolic dominant state. It is logical to think though, that growth will be compromised at higher water speeds than those tested here, as has been found in salmonids [30,31] and other species, such as striped bass *Morone saxatilis*[32]. Indeed, routine gut blood flow, which is a basic requirement for effective digestion, is reduced in salmon as they swim progressively faster and can stop with abrupt stresses [33,34].

Another effect that may contribute to exercise-induced growth is the possibility of salmon juveniles changing from active to passive (ram) ventilation. Ram ventilation is the capacity of some fish species to ventilate passively by opening their mouths when swimming or facing high water currents, allowing water to pass through the gills with enough pressure for gas exchange to occur without the need for active branchial pumping [35,36]. The energy sparing effect of ram ventilation ranged from 8.4 to 13.3% in adult rainbow trout, which shifted ventilation mode when swimming above 0.5-1 BLs^-1^[37]. Nevertheless, we cannot know for certain if this is also the case in this study.

## Conclusions

This study provides the first evidence demonstrating that inherent swimming performance in juvenile fish may predetermine disease resistance later in life. Fish classified as good swimmers showed a significantly lower mortality when challenged with IPN than fish classified as poor swimmers. Our results further suggest that the inherently poor swimmers are more sensitive to the intensity and design of the training regimes than good swimmers. Finally, the results confirmed that sustained exercise at high intensity stimulates growth performance of Atlantic salmon, while exercise at lower intensities has less effect.

The great variability in swimming performance within populations of fish opens up novel possibilities for phenotype or marker-assisted selection in breeding programs and as a discrimination tool to sort out poor juvenile fish when it is still cost-effective.

## Methods

### Experimental fish

Juvenile Atlantic salmon belonging to the Salmobreed strain were produced and reared at Nofima AS, Sunndalsøra, Norway. Freshwater stage procedures took place at the same research station, which is an approved facility under the Norwegian Animal Research Authority (NARA). Stunning and sampling of fish was done in agreement with the Norwegian regulations. As fish were exposed to different sustainable water velocities that did not induce any obvious stressful state, no specific NARA approval was required according to Dr. G. Baeverfjord, member of the NARA board and local NARA officer at Nofima AS.

### Swimming performance screening and training experimental setup

**Figure 5:**
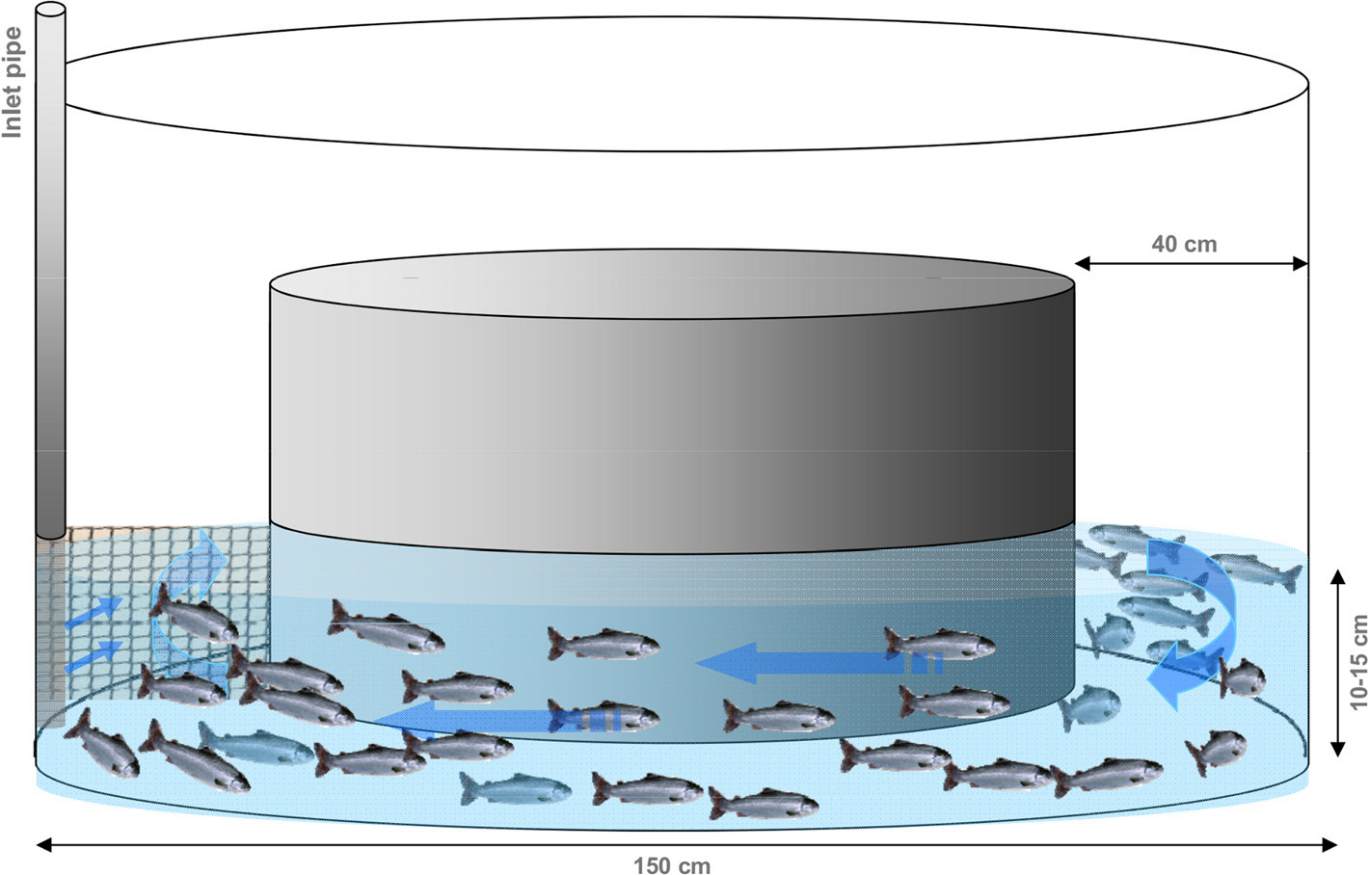
**Swimming performance screening test.** Groups of approx. 100 fish were placed in the screening tank each time and allowed to acclimate for 15 minutes at minimum water speed before start of the water speed increments. A plastic mesh (left side) prevented fish from drifting backwards. Inner ring was lifted one centimetre with the help of PVC pieces (not on diagram) to allow water drainage into the centre of the tank where the main outflow was located. Blue arrows indicate direction of water flow (clockwise).

A total of 1355 fish were individually tagged (Passive Integrated Transponder (PIT), Glass tag Unique 2.12 × 12 mm, Jojo Automasjon AS, Sola, Norway) and measured (mass ± S.D. = 40.7 ± 0.2 g and fork lengths = 15.0 ± 0.3 cm) before being graded according to their swimming performance. Groups of approximately 100 fish were placed in a pre-conditioned 1.5 m diameter circular tank with an inner ring to reduce the swimming area to a 40 cm radius (Figure 5). The water inlet to the swimming area was tangentially situated on the side of the outer tank so that it generated the maximum water velocity. The inner ring was placed on four pieces of PVC (1 cm high) that allowed the water to drain freely to the center of the tank, while preventing the fish from leaving the swimming area. Maximum water inflow generated water velocities of 42–20 cm s^-1^ nearest the center, 73–58 cm s^-1^ in the middle of the stream and 97–81 cm s^-1^ furthest from the center. A grid (painted metal meshing) was secured downstream of the water inlet to prevent fish from drifting backwards, and a floodlight placed above the grid encouraged fish to remain upstream. Water velocity and height (10–15 cm) were controlled by adjusting the water supply valve and the position of the draining stand pipe. After being introduced into the swimming flume, fish were left undisturbed for 15 min at the lowest speed to acclimatize. Water speed was then increased gradually every 1–2 min until half of a fish group had been removed from the tank. Fish that were unable to swim against the increasing water current typically laid against the back-mesh grid. They were removed with a dip-net, identified (PIT tag reading) and placed back in their rearing tank. During the trial, fish were regularly and gently repositioned to ensure that they would all be exposed to testing conditions and would not evade from the high speed outer portion of the swimming ring. Based on their swimming performance, fish were then allocated to one of two groups. The first 50% that stopped swimming were categorized as “poor swimmers”, and the last 50% still swimming were the “good swimmers”. Both poor and good swimmers were randomly mixed among 16 cylindro-conical experimental tanks (500 l, 82 cm in diameter, 77–86 fish tank^-1^) and left undisturbed for one week before the start of the training regimes. The center of each experimental tank was fitted with a plastic pipe (31.5 cm diameter), which reduced the area in the tank with lowest water speed. A frequency-controlled pump (Hanning Elektro Werke, PS 18–300; Oerlinghausen, Germany) directed the water current and a wire mesh fence, attached between the pipe and the edge of the tank, prevented the fish from drifting backwards. The water speeds were calibrated by using the average speed measured at twelve points in the tank (four horizontal locations and three depths at each location (Höntzsch HFA propeller, Waiblingen, Germany with HLOG software)). Five different sustained exercise-training regimes were tested; the control regime in quadruplicate tanks and the other regimes in triplicate tanks. Three of the training regimes were continuous velocity: the control (C; 5.7 cm s^-1^), medium intensity (M; 11.5 cm s^-1^) and high intensity (H; 23 cm s^-1^) regimes. At start of the 10-week training experiment, these speeds were equivalent to 0.38, 0.77 and 1.53 BLs^-1^ for C, M and H, respectively. As fish grew during the trial, average relative water speeds were reduced to 0.32 (C), 0.65 (M) and 1.31 (H) BLs^-1^. The two remaining training regimes used interval training, with daily increments in the relative water velocity from 0.32 to 1.31 BLs^-1^ for either a single 8 h period (Long interval; Lint) or four 2-h periods for a total high speed training period of 8 h (short interval; Sint). Theoretically, both interval groups swam the same distance as the M group. The 10 weeks of training were followed by a one-week recovery at control speed prior transferring the fish to seawater. To stimulate smoltification during the experiment, fish were exposed to a short daylight regime (12–12 Light–dark) for the first six weeks, followed by continuous light for the remaining five weeks. Measurement of ATPase in gills (n = 10) sampled from each group was conducted in a commercial laboratory, Havbruksinstituttet AS, Bergen, Norway, and confirmed that all fish were sampled within the smolt-window (data not shown). Water temperature was measured daily (10.5 ± 0.8°C) while oxygen saturation was measured weekly and was maintained over 85% with oxygen supplementation. Dead fish were removed with daily inspections and weighed.

### Growth and feed intake

During the training experiment, fish were fed to excess an extruded diet based on fish meal, ground wheat and fish oil (produced at Nofima AS, Fyllingsdalen, Bergen, Norway), using automatic feeders. The effluent water of each tank was led into a wire mesh box to enable sieving of waste feed. To minimize feed leaching, the effluent water was directed to two different areas of the wire box using pinch valves on the water pipes, dependent on whether feeding was occurring. The waste feed, expressed as dry matter (DM) content, was used to recalculate daily feed intake in order to adjust ration level every second day (Helland et al., 1996). After the change in the photoperiod at the sixth week of training, feeding regime was increased from 12 (6.25 min h^-1^) to 24 (3.12 min h^-1^) times per day.

Fish were weighed in bulk after six weeks of training and a two-day fast. At the end of the trial, fish were individually re-weighed and re-measured.

### Viral challenge test

Following the 1-week detraining period, approx. 110 fish per training regime and training control, including both performance groups, were pooled and transferred to seawater at VESO research station (Vikan, Norway) for an IPN challenge test. An additional nine fish per training regime and training control groups were similarly pooled and transferred to act as infection controls (unchallenged fish). The fish to be challenged as well as those acting as infection controls were acclimatized for one week in a separate 1.5 and 1 m^3^ tank (11 ± 0.2°C and 0.5 l kg^-1^ min^-1^; water volumes were adjusted to achieve similar densities). The IPN challenge test was performed by co-habitation and started when 20% of IPN-infected challenger fish were added to the experimental tank. A similar proportion of uninfected smolts were added to the unchallenged tank, keeping similar densities in both tanks. Challenger fish were previously marked by a fin clip and injected with 5 ml of ~3 × 10^6^ TCID_50_/ml of IPNV (strain V-1244 cultured at the Norwegian School of Veterinary Science). Throughout the challenge test, fish were observed and mortalities were recorded daily for 45 days, when the trial was terminated. A representative selection of dead fish was confirmed positive for IPNV and was further bacteriologically examined on 2% NaCl blood agar plates as part of a standard procedure during the challenge at VESO. All trials at VESO were performed according to Norwegian regulations for care and use of fish in research as stated in the Agriculture and Food Department regulation FOR 1996–01–15 Nov. 23.

### Gene expression analyzes

Fish used for gene expression analyses were sampled at the end of the IPN challenge test (day 45), when mortality of all groups had leveled off. Challenged fish belonging to both swimming performance categories (poor and good swimmers) were sampled for each of the regimes C, M and Lint, while nine unchallenged control trained (C) fish were used as hybridization controls for microarray analyses. Cardiac ventricle and head kidney tissues were immediately dissected from fish killed by a blow to the head and stored in RNA*later* (Ambion, Austin, TX, USA). Total RNA was extracted using TRIzol and purified with PureLink RNA Mini Kit (Invitrogen, Carlsbad, CA, USA) following manufacturers guidelines. RNA concentration was measured using NanoDrop 1000 spectrophotometer (Thermo Fischer Scientific, Waltham, MA, USA), and RNA integrity was assessed with an Agilent 2100 Bioanalyzer (Agilent Technologies, Waldbronn, Germany). All samples had a RNA Integrity Number (RIN) above 9.

Microarray analyses were performed with the salmonid oligonucleotide microarray (SIQ2.0, NCBI GEO platform GPL10679), consisting of 21 K features printed in duplicate on 4 × 44 K arrays from Agilent Technologies [38]. Eighteen two-color microarray hybridizations were performed. Individual heart ventricle samples of challenged fish from poor and good swimming performers (n = 9 each; 3 from each training regime) were competitively hybridized against a pooled sample consisting of equal amounts of RNA from the infection controls (n = 9) per array. Unless specified otherwise, all reagents and equipment were from Agilent Technologies and used according to manufacturer’s protocol. Amplification and labeling of RNA (200 ng) were done using the LowInput-QuickAmp Labeling kit. Cy5 (test) and Cy3 (control) labeled RNA was purified with RNeasy Mini Kit (Qiagen, MD, USA), and the Gene Expression Hybridization Kit was used for RNA fragmentation. Hybridizations were performed for 17 h at 65°C and rotation speed of 10 rpm in a hybridization oven. Arrays were washed twice (Gene Expression wash buffers 1 & 2) and immediately scanned with a GenePix 4100A (Molecular Devices, Sunnyvale, CA, USA) at 5 μm resolution and with manually adjusted laser power to ensure an overall intensity ratio close to unity between Cy3 and Cy5 channels and with minimal saturation of features. The GenePix Pro 6.1 software was used for spot-grid alignment, feature extraction of fluorescence intensity values and spot quality assessment. Low quality spots were filtered with aid of GenePix flags as well as by the criterion (I-B)/(S_I_-S_B_) ≥0.6 where I and B are the mean signal and background intensities, respectively, and S_I_ and S_B_ are the respective standard deviations. Data were exported into the STARS platform [38] for data transformation and Lowess normalization of log_2_-expression ratios (ER). Differentially expressed genes were selected based on spot signal threshold, log_2_-ER average > |0.6| in at least two individuals and significant difference between groups (swimming performers) p < 0.05, n = 9 (Student’s *t*-test). Recording of microarray experiment metadata was in compliance with the Minimum Information About a Microarray Experiment (MIAME) guidelines (Brazma et al., 2001). Microarray results were submitted to GEO (GSE38603).

Expression of single genes (VRGs) was assessed by quantitative real-time RT-PCR (qPCR) on heart ventricle and head kidney samples from swim performers of the three groups C, M and Lint at day 45 post-challenge. Expression levels were calculated relative to expression levels of the same nine infection control samples used for microarrays. A total of six and eight VRGs were analyzed for heart (n = 16 per regime; 8 good and 8 poor swimmers) and head kidney (n = 10 per regime; 5 good and 5 poor swimmers), respectively. The same samples from both tissues were further scanned for viral presence by using a set of specific IPNV primers (Table 2). Confirmation of array results by qPCR was based on eight genes (4 up- and 4 down-regulated) in the same individual samples as used for microarrays.

**Table 2:** List of primers used for the qPCR reactions

**Genes**	**Short name**	**Sequence 5' to 3'**	**Accession**
*radical S-adenosyl methionine domain-containing protein 2*^*1*^	Rsad2	F-GTACCGCAGATGCACAACAC	AF076620
		R-TTGACACTGCTTGGAGTTGC	
*interferon inducible protein Gig1*^*1*^	Gig1	F-GGCAACCTGAATCCAGAAGA	DW569595
		R-GTCTGGACGCAGACTGATGA	
*VHSV-inducible protein*^*1*^	Vhsv2	F-GGTGAAGACCTGGACCTGAA	BT072288
		R-TGACCCCTGTTGACCTTCTC	
*interferon-induced protein with tetratricopeptide repeats 5*^*1*^	Ifit5	F-CAGAGAGGTGCCAGGCTAAC	BT046021
		R-TGCACATTGACTCTCCTTGG	
*interferon induced with helicase C domain 1*^*1*^	Mda5	F-CAGAGGTGGGGTTCAATGAT	NM001195179
		R-AGCTCGCTCCACTTGTTGAT	
*DEAD/H (Asp-Glu-Ala-Asp/His) box polypeptide RIG-I*^*1*^	Rig-I	F-GACGGTCAGCAGGGTGTACT	DY714827
		R-CCCGTGTCCTAACGAACAGT	
*barrier-to-autointegration factor*^*1*^	Baf	F-ACAGACCCCTCATCATCCTG	BT049316
		R-CGGTGCTTTTGAGAAGTGGT	
*signal transducer and activator of transcription 1 isoform alpha*^*1*^	Stat1a	F-CGGTGGAGCCCTACACTAAG	CB513054
		R-GGGATCCTGGGGTAGAGGTA	
*interferon-inducible protein Gig2-like*^*1*^	Gig2	F-GATGTTTCATGGCTGCTCAA	BT044026
		R-CTTTTCGGATGTCCCGACTA	
*B-type natriuretic peptide*^*2*^	BNP	F-TCGACAAATCCGCAATAAGA	CK883650
		R-TTGAGCCAATTCGGTCTAGC	
*putative collagen alpha 1*^*2*^	put_Coll-a1	F-AACCCTGAACCCCTCAGTCT	CA038317
		R-TGGTCCTACCGTCTGGTTTC	
*leukocyte elastase inhibitor*^*2*^	LEI	F-TCTCAGATGGCAAAGGCTCT	BT045959
		R-GTTGGCCAGTTTCAGGATGT	
*elongation factor 1a*^*3*^	EF1α	F-CACCACGGGCCATCTGATCTACAA	BT072490
		R-TCAGCAGCCTCCTTCTCGAACTTC	
*18S rRNA*^*3*^	18S	F-GCCCTATCAACTTTCGATGGTAC	AJ427629
		R-TTTGGATGTGGTAGCCGTTTCTC	
*infectious pancreatic necrosis virus_polyprotein*^*4*^	IPNV	F-CCGACCGAGAACAT	AJ877117
		R-TGACAGCTTGACCCTGGTGAT	

^1^ Virus-responsive genes (Figure 4).

^2^ Genes used for microarray validation.

^3^ Reference genes used for normalization.

^4^ IPNV gene used for relative virus quantification (described in Results section).

All qPCR primers were designed using the ePrimer3 from the EMBOSS online package [39], except for the IPNV primers [40] and synthesized by Invitrogen (Table 2). Synthesis of cDNA was performed on 0.5 μg of DNAse treated (DNA-free; Ambion) RNA samples using TaqMan^@^ Reverse Transcription reagents (Applied Biosystems, Foster City, CA, USA) and primed with an equal mix of oligo dT and random hexamers. PCR reactions were prepared manually and run in duplicates in 96-well optical plates on a LightCycler® 480 (Roche Diagnostics, Mainheim, Germany) using 2X SYBR Green Master mix (Roche), 5 μl of cDNA samples and a primer concentration of 0.42 μΜ each in a final volume of 12 μl. For all genes, cDNA was previously diluted 1:10 (1:1000 for 18S). qPCR thermal cycling was as follows: 5 min pre-incubation at 95°C, followed by 45 amplification cycles consisting of 95°C for 10 s, 60°C for 15 s and 72°C for 15 s, followed by a melting curve protocol (95°C for 5 s, continuous increase from 65°C to 97°C) to assess specificity of the amplicon. Fluorescence was measured at the end of every extension step and throughout the melting curve step. Cycle threshold (C_T_) values were calculated using the second derivative method. Duplicate reactions differing more than 0.5 C_T_ values were discarded, and values were averaged for relative quantification. PCR efficiency was assessed by six 10-fold serial dilutions of pooled sample templates for each primer pair. Relative expression ratios were calculated by the Pfaffl method [41] with normalization against two reference genes (*18S* and *Elongation factor 1α*). An index value of VRG expression was calculated for each group (training regime or swimming performance) by averaging the relative expression ratio of the single genes.

### Calculation and statistics

Relative feed intake: 100 × (dry feed intake/mean body mass (BM)/days fed).

TGC: 1000 × [(BM_1_^0.33^ – BM_0_^0.33^)/∑day-degrees], where BM_1_ and BM_0_ are final and initial body masses, respectively.

FER: (Wet fish gain + dead fish mass)/dry feed intake.

CF: 100 × BM (g) × fork length (cm)^-3^.

For growth and CF analyses, the individual fish data were analyzed by analysis of variance in a hierarchical model including the fixed effect of training regime and the random effect of tank within regime. The mean data for each tank were tested by variance analysis (means compared using the least-squares means procedure) (SAS software, version 9.1, SAS Institute, Inc., Cary, NC, USA). Percentage data were transformed (arcsine square root) before being subjected to analysis. Differences between training regimes were considered significant at the p < 0.05 level, and are presented as mean ± SEM.

Differences in survival during the IPN challenge test were evaluated using the Mantel-Cox test in GraphPad Prism (version 5.01, GraphPad Software, Inc., San Diego, CA, USA). For the microarray analyses, expression differences between the groups where assessed by Student’s *t*-test; p < 0.05, and data are presented as log_2_ER ± SEM. Difference in expression levels for the indexed values of pooled VRGs, was assessed by paired Student’s *t*-test, p < 0.05; between target and control groups in qPCR. Correlation between microarray and qPCR results for selected genes was assessed by Pearsons’ *r*.

## Competing interests

The authors declare that they have no competing interests.

## Authors’ contributions

VC carried out the molecular studies, participated in samplings, data interpretation and drafted the manuscript. BG performed the growth and nutrition studies analyses. SMJ interpreted the microarray data and drafted the corresponding section. JH participated in the design of the study and revised the manuscript. GC designed the screening test facilities, performed the classification of fish according to swimming capacities, and revised the manuscript. APF participated in designing the study and critically revised the manuscript. AK analyzed the microarray data and revised the manuscript. SJH designed the fish training facilities and performed and participated in growth analyzes. HT obtained the funding, conceived and designed the study, coordinated and participated in samplings, data interpretation and drafting of the manuscript. All authors have read and approved the final manuscript.
